# A Review on Long COVID Screening: Challenges and Perspectives Focusing on Exhaled Breath Gas Sensing

**DOI:** 10.1021/acssensors.4c02280

**Published:** 2024-12-16

**Authors:** Lorena Díaz de León-Martínez, Gabriela Flores-Rangel, Luz E. Alcántara-Quintana, Boris Mizaikoff

**Affiliations:** † Institute of Analytical and Bioanalytical Chemistry, Ulm University, Albert-Einstein-Allee 11, 89081 Ulm, Germany; ‡ Breathlabs Inc., Spring, Texas 77386, United States; § Unidad de Innovación en Diagnóstico Celular y Molecular, Coordinación para la Innovación y la Aplicación de la Ciencia y Tecnología, Universidad Autónoma de San Luis Potosí, Av. Sierra Leona 550, Lomas 2a sección, 78120, San Luis Potosí, México; ∥ Hahn-Schikard, Sedanstrasse 14, 89077 Ulm, Germany

**Keywords:** long COVID, screening, diagnosis, exhaled breath, VOCs, sensors, sensing, electronic nose, AI-driven-chemometrics

## Abstract

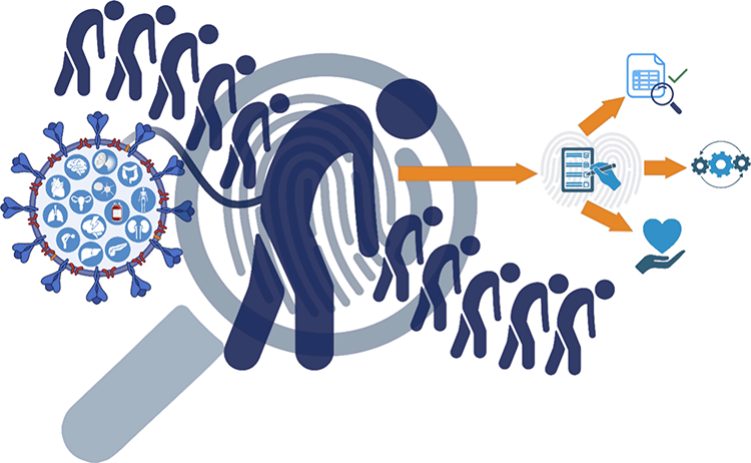

Long COVID (LC) is a great global health concern, affecting individuals recovering from SARS-CoV-2 infection. The persistent and varied symptoms across multiple organs complicate diagnosis and management, and an incomplete understanding of the condition hinders advancements in therapeutics. Current diagnostic methods face challenges related to standardization and completeness. To overcome this, new technologies such as sensor-based electronic noses are being explored for LC assessment, offering a noninvasive screening approach via volatile organic compounds (VOC) sensing in exhaled breath. Although specific LC-associated VOCs have not been fully characterized, insights from COVID-19 research suggest their potential as biomarkers. Additionally, AI-driven chemometrics are promising in identifying and predicting outcomes; despite challenges, AI-driven technologies hold the potential to enhance LC evaluation, providing rapid and accurate diagnostics for improved patient care and outcomes. This review underscores the importance of emerging and sensing technologies and comprehensive diagnostic strategies to address screening and treatment challenges in the face of LC.

Long COVID (LC) is a complex and heterogeneous clinical syndrome characterized by the persistence of a diverse range of physical and neurological symptoms and impairments that continue beyond the acute phase of the SARS-CoV-2 infection. These symptoms and impairments manifest in individuals who have recovered from the initial acute phase of COVID-19, and they often endure for a period extending from weeks to months following acute infection. LC encompasses a spectrum of clinical manifestations, which may affect multiple organs and systems.^1^ LC must be differentiated from post-COVID (PC), as LC covers long-term health problems, which last beyond the acute illness phase of 4 weeks after the infection, and PC refers to symptoms that are still present even after 12 weeks or arise newly or again after an infection and cannot be explained otherwise.

While a precise calculation of LC prevalence is still emerging, it is currently estimated that at least 39 million people worldwide have LC; this is calculated considering an incidence estimate that indicates 10% of people infected with the SARS-CoV-2 virus have this condition based on the number of documented cases to date. However, this figure is expected to be higher due to undocumented cases.^2^ The estimated reported incidence by group is (i) 10–30% of nonhospitalized cases, (ii) 50–70% of hospitalized cases, and (iii) 10–12% in vaccinated people. Even though LC affects all genders at all ages, a recent study by the IHME reports that women are twice as likely as men to develop the disease;^3^ in addition, an estimate of the proportion of people with at least one of the three most reported symptoms of this condition (persistent fatigue with bodily pain or mood swings; cognitive problems; or ongoing respiratory problems) is presented, among other significant findings. This condition’s symptoms vary and affect several organs and systems simultaneously. Among the most documented symptoms are cardiovascular, thrombotic, and stroke conditions, chest pain, palpitations, chronic cough, persistent respiratory problems, headache, dyspnoea, persistent fatigue, abdominal pain, memory loss, sleep disorders, depression, anxiety, loss of smell, muscle aches, stomach aches, diarrhea, tinnitus, skin rashes, reproductive system dysfunctions, and cognitive impairment.^4,5^

The etiology and pathophysiology of LC have not yet been fully elucidated, and its clinical presentation may vary widely among affected individuals. Nevertheless, a proposal on how the pathophysiology of this condition works is stated in work by Iwasaki and Putrino, 2023.^6^ The available data implicate the multisystemic nature of COVID-19, immune dysregulation, autoimmunity, viral persistence, virus reactivation, and inflammation-triggered chronic changes. The condition presents significant challenges for diagnosis, management, and healthcare delivery, underscoring the need for further research to understand its underlying mechanisms and to develop effective screening, diagnostic, and treatment strategies. Establishing standardized definitions, diagnostic criteria, and surveillance systems remains a critical challenge for healthcare authorities worldwide. Evidence-based measures are essential for diagnosing, monitoring, and managing symptoms, which will support the development of standardized surveillance infrastructure and effective guidelines for assessing LC. It is reported that more than 7 million quality-adjusted life years might be lost due to the condition and that its prevalence may be reducing the workforce by nearly 3 million workers (this is only considering the OECD countries), adding to dramatic economic costs, including treatment, activities limitations, reducing participation in labor, and indirect and direct medical costs, among others.^7^

More than 22 countries worldwide have set up dedicated LC clinics, demonstrating that primary care has a key role in healthcare systems to treat this condition. Nevertheless, the demand for LC services in a multidisciplinary approach for its broad symptomatology is increasing and appears to exceed supply availability. One of the most critical points in primary care is a timely and accurate diagnosis. Currently, LC screening and diagnosis are performed based on different methodologies depending on the symptomatology reported by the patient. Among these, are patient symptom questionnaires, which are designed to help healthcare providers and researchers evaluate and document the symptoms and health status of individuals experiencing LC; other diagnostic tests to assess further symptoms include blood tests,^8^ pulmonary function tests,^9^ chest imaging,^10^ echocardiogram,^11^ cardiac MRI,^12^ functional tests (Functional 6MWT, STS, SPPB),^13^ cardiopulmonary stress testing,^14^ imaging,^15^ and others. In this regard, different guidelines focused on diagnosing and managing this condition have been developed by several international and clinical organizations worldwide, including the ESCMID, NICE, SIGN, and RCGP guidelines. These guidelines recommend healthcare professionals caring for people with suspected or confirmed acute COVID-19 who present to any healthcare facility regardless of whether they have been hospitalized or have had a positive or negative SARS-CoV-2 test result. The guidelines emphasize providing information so that people understand their symptoms and know when to seek help. Despite all these diagnostic tools, LC is a very complex disease to diagnose; in this regard, the growing need for diagnosis of this condition has led researchers around the world to develop new criteria and diagnostic tests to include the different symptoms to ensure a timely diagnosis and therefore a better outcome in implementing the correct treatment for the associated symptoms.^6^ Despite the available tools for assessing LC symptoms, diagnosing and managing the disease remain significant challenges for healthcare professionals and, consequently, for those suffering from it. The wide range of symptoms, lack of validated diagnostic biomarkers, inconsistent recognition and awareness, limited treatment options, and resource constraints—such as the limited specialized clinics, rehabilitation centers, and access to multidisciplinary teams and specialized equipment—make the LC landscape even more complicated. In light of this, there is a growing need for the development of cutting-edge technologies that can assist healthcare professionals in accurately diagnosing and managing LC while also simplifying this complex landscape and offering new tools for effective long-term care.

In this regard, the rise of wearable sensor technology has the potential to be a game-changer in the management of LC. These sensors enable continuous, non-invasive, real-time monitoring of vital signs and other physiological parameters, offering invaluable insights into the persistent symptoms that define LC, such as fatigue, heart rate irregularities, and respiratory issues. By tracking changes over extended periods, wearable sensors allow healthcare professionals to better understand the fluctuating nature of symptoms, providing personalized care and early detection of exacerbations. This real-time data collection, which includes heart rate variability, oxygen saturation, and respiratory rate, could potentially be crucial in tailoring treatments, in-house monitoring, and improving outcomes for LC patients.^16^

Electronic noses (eNoses) represent an innovative subset of wearable sensor technology with promising applications in LC monitoring. These devices detect patterns in volatile organic compounds (VOCs) present in exhaled breath, generating a “breath-print” that can reflect metabolic changes related to respiratory or systemic disease. The novelty of eNoses lies in their ability to non-invasively capture complex breath patterns that may be indicative of ongoing inflammation, lung damage, or metabolic dysregulation seen in LC. As VOC profiles can dynamically change with disease progression, eNoses offer the potential for real-time, at-home monitoring of LC patients, facilitating early interventions when adverse changes in breath profiles are detected. This personalized approach, grounded in continuous breath analysis, makes eNoses a promising venue for improved patient care and management.^17^ The integration of artificial intelligence (AI)-driven chemometrics in eNose data analysis has significantly enhanced the accuracy and utility. Chemometrics, which involves the application of mathematical and statistical methods to chemical data, is essential for interpreting the complex VOC patterns captured by eNoses.^18^ With the addition of AI these analyses become more advanced, enabling the identification of subtle patterns and correlations that might otherwise go undetected. Machine learning (ML) algorithms can classify breath-prints, track disease progression, and have the potential to predict symptom flare-ups with high precision. Given the multisystemic nature of LC and the complexity in the identification of symptom patterns, this enhanced approach—combining exhaled biomarker analysis integrated with AI and patient clinical history—has the potential to not only improve diagnostic accuracy but also opens the door to predictive and preventive healthcare solutions. This could assist the healthcare professionals in accurately managing the disease, enabling informed decisions and better treatment strategies, providing regular patient follow-up, and hence improving the quality of life of those suffering from LC.

Therefore, in the present scenario, this review is focused on presenting an overview of sensor technologies, particularly eNoses, in the context of long-term COVID management. As LC continues to impact millions globally, the need for advanced, non-invasive diagnostic tools is growing, and this technology has the potential to enhance diagnostic precision while offering real-time monitoring and personalized care for LC patients. This review will explore the current methodologies, focusing on the innovative application of sensor-based technologies while addressing the challenges and future perspectives in AI-driven LC assessment.

## Sensing Long COVID

The current diagnostic and management of LC faces many challenges, such as self- and indiscriminate medication consumption, inadequate support and limited access to information about LC and treatment, the lack of institutions dedicated and trained to diagnose, treat, and monitor this ailment, timely detection, and thus the frustration from the patients when not being diagnosed accurately due to the multisystemic nature of the disease. The impact of LC has transformed the approach to disease management, shifting the focus toward individualized healthcare monitoring. In this sense, there is an increasing need for LC patients to self-monitor vital organs and to record on a regular basis. Individuals should remain vigilant regarding any decline in their physical condition and consistently communicate any health concerns to their healthcare providers. Consequently, it is essential to integrate wearable devices into daily life to monitor health parameters effectively and offer real-time medical insights to individuals.

Wearable devices have played a crucial role in this shift by enabling continuous tracking and monitoring of physiological parameters in the human body. These devices are particularly advantageous for the early detection of COVID-19 (including asymptomatic and presymptomatic cases), real-time monitoring of patient conditions, and ongoing surveillance of individuals recovering from COVID-19 and now in the face of LC, as well as healthy individuals, to enhance management strategies. A wide range of wearable devices is now available to remotely monitor various physiological parameters of the human body, allowing for both patient and self-monitoring. Examples include smartwatches, smart tattoos, rings, smart facemasks, nanopatches, and more, which are designed to monitor key physiological indicators.^16,19^

In the wearable device design, sensors play a key role in monitoring human body parameters such as body temperature, respiration rate, heart rate, oxygen levels, sleeping patterns, etc. The integration of wearable sensors with biomarkers provides considerable advantages over traditional sensors and devices. These advantages include (i) non-invasive and remote capture of clinical data, (ii) prompt access to digital data for clinical applications, (iii) widespread availability and use of cell phones and smartwatches, (iv) the integration of AI for pattern recognition and predictive algorithms, (v) online surveillance from healthcare providers to assist them in decision making, and (vi) enhancement of the telemedicine approach, among others.

**Figure 1:**
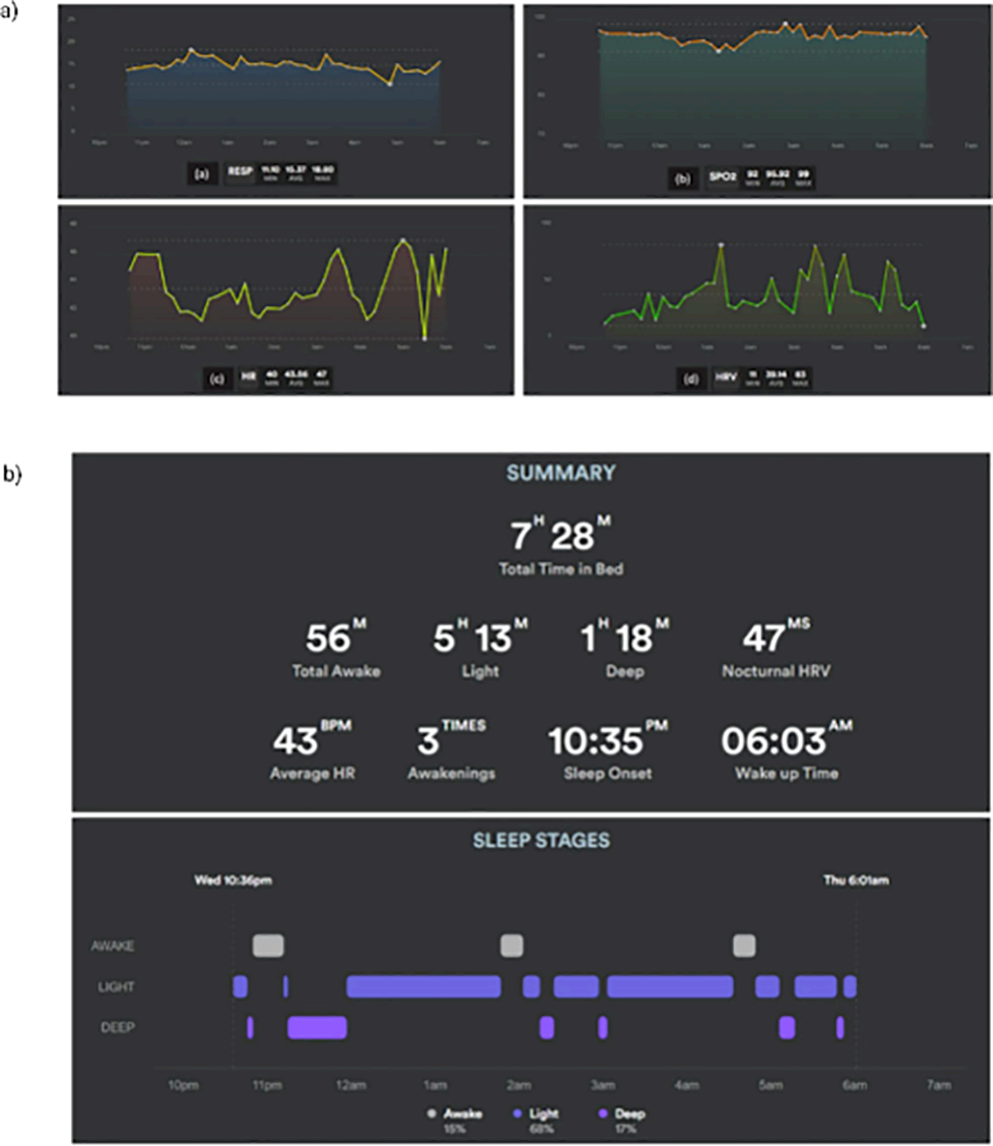
(a) Recording example of biometrics during the night for a patient with long COVID-19. (a) RESP: respiratory rate (respirations per minute); (b) SpO2: saturation of oxygen (%); (c) HR: heart rate (beats per minute); and (d) HRV: heart rate variability (beats per minute). (b) Recording examples of sleep summaries and sleep phases during the night for a patient with long-term COVID-19. HR: heart rate; HRV: heart rate variability. Reproduced from Mekkhael et al. (2022), https://www.jmir.org/2022/7/e38000/ distributed with Creative Commons Attribution License (https://creativecommons.org/licenses/by/4.0/).

At the time of the study, there were only a few publications related to LC assessment through sensors, as mentioned in Table 1. From the technical studies, Mekhael et al. (2022) used a wrist wearable sensor (Biostrap https://biostrap.com/) to monitor sleep quality through heart rate, heart rate variability, oxygen saturation, and respiratory rate in LC patients (Figure 1a,b). They correlated these values with deep sleep and altered sleep behavior and demonstrated that patients with LC presented altered sleep when compared to controls.^20^ Sun et al. (2024) reported the application of a wearable sensor to monitor myalgic encephalomyelitis/chronic fatigue syndrome (ME/CFS) attached to the ankle of LC patients and correlated the ME/CFS with the steps and the time that the person stays up in a day. Their results suggest that the ME/CFS can be correlated with LC severity.^21^ Finally, Armstrong et al. (2023) monitor chest and diaphragmatic breathing patterns in LC patients; nevertheless, they still do not present results related to the patient outcome.^22^

**Table 1:** Publications about Sensors Applied to Long COVID Assessment

Reference	Sensor	Biomarkers	Type of publication
(16)	N/A	N/A	Review
(23)	N/A	N/A	Review
(24)	N/A	N/A	Review
(21)	Wearable sensor	Myalgic encephalomyelitis/chronic fatigue syndrome	Research/Preprint
(25)	Lab-on-a-chip	N/A	Review
(22)	Respiration Sensor	Chest and diaphragmatic breathing patterns	Conference letter
(20)	Wearable sensor	HR, HRV, respiratory rate (RR), and oxygen saturation (SpO2)	Research
(26)	Wearable sensor	Breathing pressure	Research
(27)	N/A	N/A	Review

While wearable sensors offer promising potential for monitoring and managing LC symptoms, their application has several limitations. One major challenge is the need for robust data privacy and security measures due to the sensitive nature of the collected health information. Additionally, there are concerns regarding the accuracy and reliability of the data generated by these devices, which can vary widely based on device quality and user compliance. Furthermore, ethical issues such as informed consent and the potential psychological impact of continuous health monitoring on patients require careful consideration. As wearable technology continues to evolve, addressing these limitations will be crucial to ensure its effective and safe use in managing LC.

## The Role of Exhaled Breath Sensing in Long COVID Assessment

Reliable technologies are urgently needed to diagnose and monitor the progression of LC, especially as the number of cases of COVID-19 continues to rise with emerging variants and associated deaths from persistent symptoms. The growing need to distinguish between seasonal illnesses and infectious diseases with similar symptoms further complicates this scenario. One of the most interesting approaches that have been proposed for LC evaluation is the exhaled breath assessment, also called “Exhalomics” (The study of VOC biomarkers in the exhaled breath).^28^

Exhaled breath is a complex biological matrix, mainly containing nitrogen (78.04%), oxygen (16%), carbon dioxide (4–5%), hydrogen (5%), inert gases (0.9%), water vapor, and volatile organic compounds (VOCs). More than 3000 VOCs have been described in exhaled breath. VOCs present in exhaled breath can be of both endogenous and exogenous origins. Endogenous VOCs are produced within the body through metabolic processes. In contrast, exogenous VOCs come from the environment, such as air, food, or other external sources, and are either exhaled unchanged or metabolized before exhalation. After inhalation and/or environmental exposures, VOCs are retained in different parts of the body depending on their breath-blood-fat partition coefficients, some of them are stored in fatty tissues and are then released to the bloodstream and exchanged into the breath through alveoli, the body even metabolizes some of them and released as other compounds; another portion is retained in the respiratory tract, enabling their combination with endogenous VOCs.^29,30^ On the other hand, the endogenous VOCs can be related to physiological and pathophysiological conditions; several tissues of the body produce these VOCs as a result of free radicals formation and lipoperoxidation in the onset of cellular damage they can either represent inflammation, metabolism, and, depending on their partition coefficient, they are excreted through the capillary-alveolar barrier from the circulation and therefore can be found in exhaled breath.^31,32^ VOC changes can be related to the current health status of an individual over a period of time, and as a result, changes in VOC absorption, metabolism, and excretion can be detected. Now that clinicians recognize distinct odors associated with infectious, metabolic, and cancerous diseases and genetic disorders, VOC profiles can serve as olfactory biomarkers to help identify life-threatening conditions and disorders. Gaining insight into the pathophysiological processes responsible for the production of disease-specific VOCs may open the way for novel therapeutic strategies across various disorders. In this regard, hundreds of VOCs have been proposed as biomarkers for diseases such as diabetes, several types of cancer, chronic respiratory diseases, and even infectious diseases. These VOCs include acetone, ammonia, nitric oxide, isoprene, methane, ethane, pentane, and aldehydes. The process by which VOCs can be detected in exhaled breath is briefly described as follows: the inhaled air travels to the pulmonary alveoli, where the excretable metabolic products diffuse into the inhaled air and are then expelled as exhaled breath. Therefore, exhaled breath must carry the fingerprint of the endogenous metabolic process, which can be associated with a pathophysiological process. Hence, they are a rich source for disease diagnosis and health monitoring.

Regarding LC, several authors have described changes in breathing patterns and exhaled breath composition associated with LC. Among these changes are breathing difficulties such as dyspnea (difficulty in breathing),^33^ altered breathing patterns (shallow or rapid breathing),^34,35^ and postexertional malaise (breathing issues exacerbated by physical activity).^36^ Increased markers of oxidative stress in exhaled breath condensate (EBC) and exhaled breath gas^37,38^ and altered nitric oxide levels (eNO) are biomarkers of airway inflammation.^39^ Other potential changes are in the VOC content in exhaled breath. Nevertheless, research that reports exhaled breath VOCs as biomarkers for LC is still scarce, and at the time of the study, there were no reports of specific VOCs that have been described that could be associated with the general health status of a person with symptoms suggestive of LC. However, some have been reported to be associated with SARS-CoV-2 virus infection, which is indicative that some of these biomarkers could be monitored during the progression of disease to recovery or even presented with prolonged symptoms. Table 2 lists VOCs reported in exhaled breath associated with COVID-19. Wilson and Forse (2023) explain some mechanisms by which SARS-CoV-2 virus infection could be related to certain VOCs reported in exhaled breath. The various studies analyzed by this research group suggest that patients with COVID-19 have higher levels of aldehydes and ketones in exhaled breath than the control subjects. In this regard, they refer that higher production of aldehydes is related to damaged tissues by inflammation processes, cytokine storms are directly associated with infection by the virus, and these generate metabolic cascades that affect various organs, causing widespread cell damage in multiple organs and systems, while causing acute inflammation in tissues and immunosuppression, leading aldehydes to travel from the site of damage to the exhaled breath where they can be detected.^40^

**Table 2:** Exhaled Breath Volatile Organic Compounds Reported for SARS-CoV-2 Infection by Different Analytical Techniques^a^

Reference	Volatile Organic Compounds Biomarkers	Analytical technique
(41)	2,3-butandione, aldehydes, 2,8-dimethyl-undecane and n-propyl acetate	MALDI-ToF-MS
(42)	Ethanol, acetone, 2-butanone, methanol, octanal, isoprene, heptanal, propanal, and propane	GC-IMS
(43)	Methylpent-2-enal, 2,4-octadiene 1-chloroheptane, and nonanal	PTR-ToF-MS
(44)	N/A	Multiplexed Nanomaterial-Based Sensor Array
(45)	Butanoate, butyraldehyde, isopropanol, acetone	GC-IMS
(46)	N/A	Electronic nose
(47)	Alcohol, acetone, carbon monoxide	Electronic nose
(48)	Octanal, nonanal, heptanal, decane, tridecane, and 2-pentyl furan	GC-ToF-MS
(49)	Formaldehyde, methanol, hydrogen sulfide, acetone, acetic acid, isopropanol, croton aldehyde, butyric acid, butanethiol	Real-time MS
(50)	N/A	Electronic nose
(51)	Benzaldehyde, 1-propanol, 3,6-methyl undecane, camphene, beta-cubebene, iodobenzene, and an unidentified compound	GC-MS
(52)	1-propanol, isopropanol, 2-(2-butoxyethoxy) ethanol, propanal and 4-(1,1-dimethylpropyl)phenol)	GC-MS
(53)	Undecane, pyridine, heptanal, dodecane, octanal, tridecane, 5-hepten-2-one, 6-methyl-, octanoic acid, methyl ester, tetradecane, 2-octenal, furfural, 2-methyl-1-hexanol, nonanoic acid, methyl ester, benzaldehyde, linalool, hexadecane, undecanal, pristane, heptadecane, lauric aldehyde dodecanal, naphthalene, dodecanoic acid, hexadecenoic acid, 5,9-undecadien-2-one, 2-phenyl ethyl alcohol, phenol, isopropyl myristate, lilial, cedrol, hexyl salicylate, methyl palmitate, isopropyl palmitate, alpha-amylcinnamaldehyde, n-decanoic acid, alpha-hexylcinnamaldehyde	GC-MS
(54)	Aldehyde, ketone, terpene, terpenoid, ester, alkane.	GC- QTOF-MS
(55)	Methylcyclopentane, benzene, octane, 2,2,4-trimethylheptane, 2,2-dimethyloctane, decene and dimethyloctaniene, decane, trimethylolethane, methyldecane, undecane, dimethyldecane and tetramethyloctane	Portable GC
(56)	Carbon dioxide, cycloheptatriene, formaldehyde, ammonium, nitrogen dioxide, carbon monoxide, acetone and ammonia	GC-IMS
(57)	Butyric acid, formaldehyde, acetone, isopropanol, hydrogen sulfide, methanol, acetic acid, croton aldehyde, and butanethiol	PTR-ToF-MS
(40)	N/A	Electronic nose

a Abbreviations: SARS-CoV-2: severe acute respiratory syndrome coronavirus 2; GC-MS: gas chromatography–mass Spectrometry, PTR-ToF-MS: Proton transfer reaction-Time of flight-Mass spectrometry; MALDI-ToF-MS: Matrix-assisted laser adsorption–desorption/ionization-time of flight-mass spectrometry; GC-IMS: gas chromatography–ion mobility spectrometry; GC-ToF-MS: gas chromatography–time of flight-mass spectrometry; n/a: not applicable or not described.

They report that ketones come from damage to the liver and pancreas derived from the infection, which alters metabolic pathways, causing ketosis, hyperglycemia, or hypoglycemia due to glucose and insulin metabolism, symptoms that undoubtedly remain present postinfection in patients with LC. Other metabolic sources of aldehydes and alkanes are derived from the oxidation of lipids and ketones in the metabolism of carbohydrates and fatty acids, which could be related to an upregulation of these molecules during and after infection. Alcohols could be associated with the metabolism of acetaldehyde in the liver, resulting in higher concentrations in peripheral blood and consequently excreted by the lungs in the exhaled breath.^40^ These are the known possible explanations for certain VOCs derived from COVID-19 infection; a likely hypothesis is that postinfection, these mechanisms are still active, derived from persistent organ damage, so they could still be present in the exhaled breath of people with LC.

Several preanalytical conditions must be considered in the study of exhaled breath, the most important being the collection of the sample itself. In this context, breath samples are typically collected using specific collection devices, which can be simple (such as disposable bags) or more complex (such as specialized breath condensers). The method of collection must minimize contamination from ambient air, ensure moisture control, and ensure reproducibility. Some approaches include (i) direct/online sampling by collecting the exhaled breath directly into a container or into an analytical device; (ii) EBC) collection, where exhaled breath is cooled to collect water-soluble compounds in a liquid form into a special device; and (iii) particle or specific compound collection by filters or sorbent materials. The standardization from the sample collection must be carried out for the analysis to be reproducible. The most commonly analyzed portion of the exhaled breath is the so-called “end-tidal air”; this refers to the portion of exhaled air that comes from the deepest part of the lungs and is the last part to leave the body during exhalation. It represents the air from the alveoli, where gas exchange between the lungs and blood occurs. Since this air is in close contact with the bloodstream, it carries the most accurate representation of volatile compounds, such as carbon dioxide and VOCs, that reflect the body’s metabolic state, and it also provides insight into a person’s respiratory function, metabolism, or presence of certain conditions. It is well-known that LC patients present different breathing patterns compared to healthy individuals, they have been related to unexplained dyspnoea following COVID-19, also changing the content of the end-tidal breath.^34,58^ Therefore, assessing the end-tidal portion of breath is critical in the present context and, when comparing the LC breath content to other respiratory conditions, also in assessing specific LC-related VOCs.

Efforts in the standardization of sampling methodologies have been extensively made; in this regard, the Task Force from the European Respiratory Society has published “The European Respiratory Society technical standard: exhaled biomarkers in lung disease”. This document provides recommendations for standardization of sample collection and evaluation of different analytical approaches for breath analysis. It explains the limitations and advantages of each exhaled breath portion when collected and analyzed, as well as recommendations for several analytic methodologies.^59^ The document emphasizes the need for standardization and the development of innovative methodologies for breath sample collection in both offline and online approaches. Additionally, it highlights the challenges associated with identifying and validating VOCs as disease-specific biomarkers for their integration into clinical practice.

Although several limitations remain in the application of VOCs in point-of-care (POC) settings, research in the exhalome field continues to evolve, demonstrating significant potential for future clinical applications.

Talking about innovation in exhaled breath analysis, several methodologies have been proposed in the literature, among which the gold standard is gas chromatography coupled to mass spectrometry (GC-MS);^60^ others include proton transfer reaction mass spectrometry (PTR-MS);^61^ selected mass flow tube mass spectrometry (SIFT-MS);^62^ field asymmetric ion mobility spectrometry (FAIMS);^63^ and time-of-flight mass spectrometry (TOF-MS),^64^ and in the last decades the use of devices based on nanomaterials has been reported; especially metal oxide semiconductor (MOX) sensors, sensors based on gold and silver nanoparticles (AuNPs, SNPs), electrochemical sensors, and some coupled with orthogonal detection technologies such as infrared spectrometry.^65^ These sensors are used in arrays in devices called electronic noses (eNose). The eNose is a device that mimics the mammalian sense of smell and is trained by pattern recognition systems to detect and classify odors. eNose systems have been used in different scenarios and are one of the most important forms of disease screening. Several reports are available on the use of eNose to monitor asthma,^66^ chronic obstructive pulmonary disease,^67^ obstructive sleep apnea,^68^ lung transplantation,^69^ lung cancer,^70^ colorectal cancer,^71^ breast cancer,^72^ infectious diseases such as tuberculosis,^73^ human rhinovirus,^74^ COVID-19^42^ and now LC.^75^ The difficulty in diagnosing this condition is an ongoing medical challenge that requires more specific information, such as disease-specific VOC biomarkers and the quantification of other important parameters. The classification of the complex biochemical processes of LC and the interactions with host metabolic pathways is likely dependent on the identification of specific biomarkers that in the near future will allow the classification of patient symptomatology even with its multisystemic nature. Continuous monitoring of the patient’s state through biomarkers is helpful in assessing progress in recovery, response to treatments, and changes in specific metabolic activities associated with particular organs to determine levels of organ dysfunction and LC effects.

Scarce are the studies regarding LC diagnosis with specific VOCs as biomarkers that are even more limited through exhaled breath analysis. However, the existing reports in the literature at the time of this review are listed in this section. In this regard, Di Gilio et al. (2023) used VOCs from exhaled breath to understand whether traces of metabolic alterations induced during the acute phase of infection are still detectable after negativization in the form of characteristic VOC patterns; they found that 5 VOCs (1-propanol, isopropanol, 2-(2-butoxyethoxyethoxy)ethanol, propanal, and 4-(1,1-dimethylpropyl)phenol) showed abundances in breath samples collected from individuals after the COVID-19 negative test. The authors discussed that traces of metabolic alterations induced during the acute phase of infection are still detectable after negativization.^52^

One approach to studying VOCs has been through medical scent detection dogs. Twele et al. (2022) trained nine dogs to detect COVID-19 and LC; the dogs managed to detect with a sensitivity of 86.7% and a specificity of 95.8% when discriminating between acute COVID-19 patients from LC patients and a sensitivity of 94.4% and selectivity of 96.1% when discriminating between control individuals and LC patients.^76^ In the same context, Grandjean et al. (2022) used the same methodology with medical detection dogs through the smell of axillary sweat. They managed to discriminate between LC patients and control individuals with an accuracy of 51.1%.^77^ This research could serve as a first step to studying and understanding VOC patterns after the infection and to propose close-patient monitoring to assess further symptoms and possibly the association with LC; these findings follow the results presented by other authors in the application of the eNose, which support the hypothesis of VOCs being present long-term after the initial infection in PC and LC patients.

**Figure 2:**
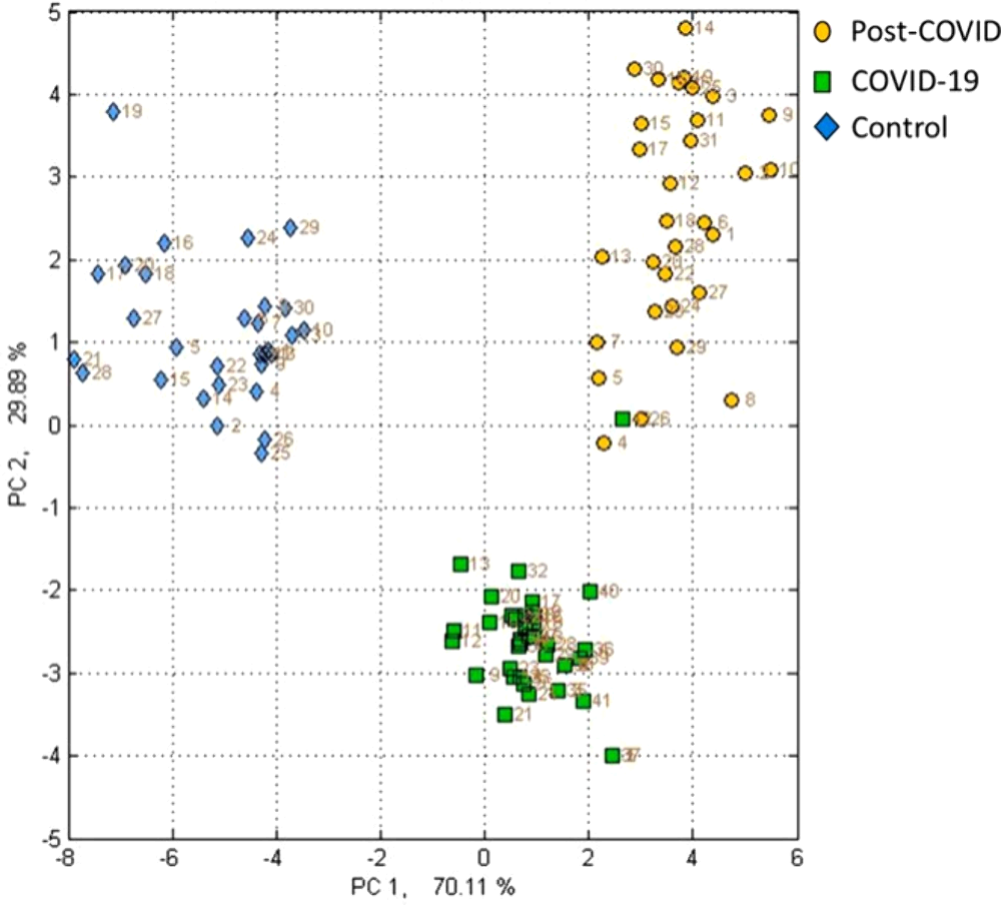
Canonical Discriminant Analysis (CDA) of the study groups. Yellow circle, post-COVID group; green square, COVID-19 group; and blue rhombus, control group. Reproduced from Zamora-Mendoza et al. (2021). https://www.sciencedirect.com/science/article/pii/S0039914021007530.

Among the few reports published on using the eNose in LC assessment, the Cyranose 320 eNose is the most widely reported. This specific eNose is one of the most used by researchers in biomedical and environmental applications; it is equipped with 32 sensors based on conductive composite technology, and the reported sensitivity of the Cyranose 320 for different gases goes from subppm levels around 0.5 to 50 ppm, considering the specificity for certain compound groups such as hydrocarbons (alkanes, alkenes, aromatic), alcohols, aldehydes, ketones, esters, acids, amines, sulfur-containing, and halogenated compounds. In this regard, Zamora-Mendoza et al. (2021) studied the global pattern of VOCs in healthy individuals through this eNose. In this proof of concept, they reported that the VOC breath fingerprint was different among healthy individuals, COVID-19-infected patients, and LC patients. They reported a sensitivity and specificity of 97.4% and 100%, respectively.^50^ One limitation of this study is that the three study groups have different demographic and clinical characteristics; nevertheless, this is expected for LC (Figure 2). The same research group provided a broader study with the same technology meant to discriminate between LC patients and healthy subjects with a larger cohort of patients; they reported sensitivity and a selectivity of 88.9 and 96.9%, respectively;^78^ the limitation of this study is that the diagnosis of LC was not based solely on impaired respiratory function. Also, there was not a validation cohort among their study groups, and the two groups also have significantly different clinical and demographic characteristics (BMI, percentage of hospitalized patients, percentage of patients who experienced a hypoxic episode, number of days on oxygen therapy, percentages of smokers, asthmatics, and patients with comorbidities much higher in the group with impaired pulmonary function), without the role of these confounding factors being excluded.

In this same context, Nidheesh et al. (2022) published a study using the same eNose to analyze VOCs during this condition; they performed match/no-match and KNN analysis tests to confirm the diagnosis of LC. Their prediction model reported 100% sensitivity and specificity,^79^ and this study compared asthma, LC, and healthy subjects. Nevertheless, they do not describe how the population was sampled and under which criteria patients were recruited and selected and also did not present a validation cohort.

**Figure 3:**
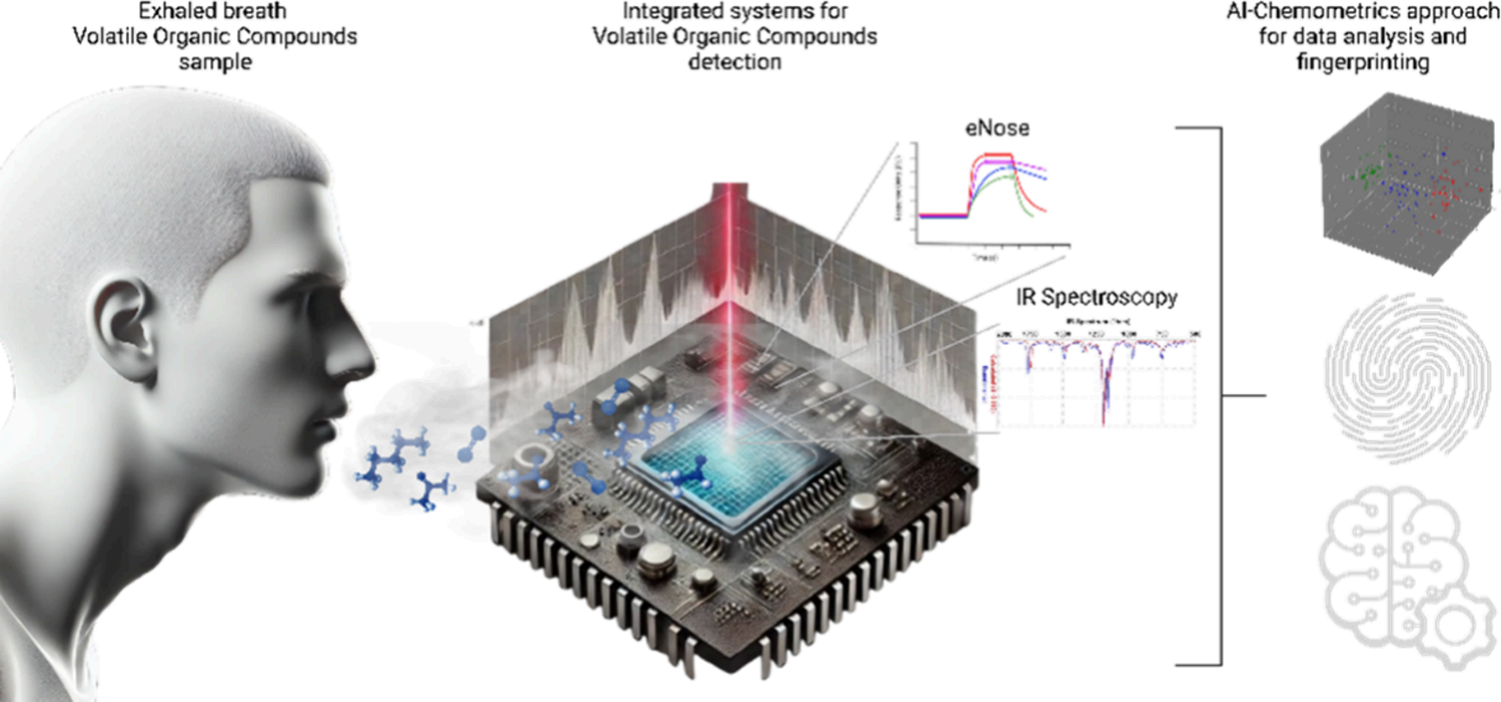
Schematic representation of gas-phase infrared spectroscopy and the electronic nose for disease screening through breath analysis.

Among other technologies with the same approach are integrated eNoses for LC. In this regard, Glöckler et al. (2023) recently proposed the development and application of orthogonal methodologies (gas-phase-infrared spectroscopy) along with eNose as a method to detect further biological fingerprints in the exhaled breath of patients with COVID-19 and as a perspective to monitor specific biomarkers in the LC context.^80^ These new generations of breath analyzers are presumed to enhance the selectivity in monitoring the progression of the infection and to evaluate the molecular changes in the exhaled breath fingerprints of these patients. This coupling improves the sensitivity and specificity of detection by cross-validating VOCs identified by the eNose through FTIR’s precise molecular analysis. Such a synergistic integration of technologies will provide healthcare professionals with a more robust and validated diagnostic methodology, strengthening their confidence in identifying and managing cases effectively (Figure 3).

Although eNose presents a potential approach in the clinical practice of LC and other disease assessments, there are several limitations that must be considered, especially in sample collection. When studying exhaled VOCs, various nondisease, patient-related factors must be taken into account, including (i) breathing maneuvers, (ii) airway caliber, (iii) dietary intake (food and beverages), (iv) alcohol and tobacco consumption, (v) physical activity, and (vi) pregnancy.^59,81^ In this regard, breathing maneuvers and airway caliber might affect the VOC concentration in either diluting or concentrating the sample and the alteration in VOCs sources region (upper vs lower airways) affecting concentration and type of VOCs sampled. The dietary intake might impact the type of VOCs present in the samples, as the type of food is metabolized, affects metabolic processes, and generates confounding VOCs (i.e., garlic, onion, or spices produce sulfur-containing compounds that are detectable in breath and can confound eNose measurements), as well as the alcohol and tobacco consumption, which directly affects the release of ethanol, benzene, toluene, and nicotine-related compounds and related metabolites, which can dominate the breath profile and interfere with VOCs patterns. Moreover, it has been reported that increased physical activity increases metabolic rates, resulting in higher production of certain VOCs like acetone and increased oxidative stress reflected in acetaldehyde and ethylene high concentrations in breath. Finally, pregnancy changes respiratory functions (i.e., increased tidal volume or expiratory flow rate) and induces significant hormonal and metabolic changes, leading to shifts in the types and concentrations of VOCs produced. Each of these factors can introduce variability into the VOC profiles detected by eNoses; these must be carefully controlled or accounted for in study design and data analysis, particularly in clinical applications where consistent VOC detection is critical.^82−85^ Also, the expiratory flow rate (EFR) can significantly impact eNose measurements, as it varies the composition and concentration of VOCs directly in exhaled breath, inconsistent flow rates across samples can lead to variability in VOC detection, and the sampling duration may result in variations, a faster EFR could generate a shorter sampling duration, potentially reducing the time available for the VOCs to be interacting with the sensors; on the other hand a slower EFR may allow more thorough interaction sensors-VOCs but could introduce biases if not standardized between patients.^81^ The airway deposition and dead space effects are also to be considered; higher flow rates may cause VOCs from deeper regions of the lungs to be less represented in the exhaled sample, whereas lower flow rates may allow more time for dead space air (air that does not participate in gas exchange) to influence the sample. This variation can lead to differences in the VOC profiles, compromising the reliability of the measurements. Finally, regarding the temperature and humidity, most eNose systems are sensitive to environmental conditions, so fluctuations in temperature, humidity, and even the humidity present in exhaled breath must be reduced to avoid sensor saturation (especially to MOX sensors), drift in the sensor response, competitive adsorption, delayed desorption, noise in the signal, and affectations in the physical properties of the sensors, leading to reduced lifetime, quality, and lack of reproducibility in the response. These challenges could be addressed by standardization of breath sampling collection, diminishing variation between patients’ EFR, temperature control to avoid humidity and condensation, the use of humidity traps, and the application of humidity sensors to measure and compensate humidity level around each measurement.^86−88^

Regarding the specific application of eNoses in LC assessment, even though the studies here are limited, these are the first steps to delve into exhaled breath analysis through eNoses for LC assessment. All of these studies potentially demonstrate the future possibility of the application of eNoses in the detection and monitoring of LC. Developing new approaches to monitoring this disease is crucial for healthcare professionals and public health authorities. These devices offer rapid and non-invasive means of diagnosing the condition, diminishing the need for extensive laboratory tests and reducing the burden on healthcare facilities, thus allowing healthcare providers to initiate targeted treatments promptly. Moreover, their user-friendly interface and automated analysis reduce the need for specialized training, enabling healthcare providers to conduct screenings efficiently. This will potentially optimize time and resources and enhance healthcare systems’ overall capacity to manage and respond to the challenges posed by LC, ultimately improving patient care and outcomes. It is important to note that these technologies are always coupled to chemometric/AI algorithms to generate fast, accurate, and reliable results. Through techniques such as principal component analysis (PCA) and discriminant analysis (DA), chemometric analysis enables the identification and classification of different odors, contributing to the development of more sophisticated and accurate systems; this approach is of great relevance in the context of diagnostics as they hold great promise in aiding healthcare professionals in decision-making and disease prognosis.

## Revolutionizing Long COVID Evaluation: The Impact of AI-Driven Chemometrics

In modern healthcare, the field of artificial intelligence (AI) has gained increasing attention in diagnostics, driven by ongoing technological advancements. The continuous evolution of research in diagnostic methods has become particularly important during the pandemic, significantly attributed to the persistent symptoms observed in LC patients.^89^ In this regard, AI presents numerous potential applications in diagnostics and screening, encompassing tasks such as image analysis,^10^ biomarker assessment,^8^ decision-making,^90^ and prognosis prediction. Its widespread adoption has proven to be useful in during the COVID-19 pandemic, underlining the versatility and effectiveness of AI in addressing complex diagnostic challenges.^91^

The literature on AI-driven chemometric applications in COVID-19 has mostly reported on three aspects: (i) the prediction of virus spread or survival rate, (ii) symptom recognition through medical images, and (iii) biomedical devices and development of drug, vaccine, and screening methodologies.^92^ In this regard, Li et al. (2020) developed a deep learning (DL) model that demonstrated excellent performance in identifying COVID-19 through chest CT scans, exhibiting a sensitivity of 90% and a high specificity of 96%. The model’s overall diagnostic accuracy, reflected in an area under the curve (AUC) of 0.96, highlights its efficiency in distinguishing COVID-19 cases. Notably, the average processing time for each CT scan was swifted at 4.51 s, further emphasizing the utility of the DL model in aiding timely and accurate diagnoses.^93^ Also, Quiroz-Juárez et al. (2021) conducted a study focused on identifying individuals at high risk early after exposure to the SARS-CoV-2 virus, employing a supervised artificial neural network (ANN). Machine learning (ML) models were trained using a combination of comorbidities, patient demographic information, and recent COVID-19-related medical data. According to the findings, the authors reported that the outcome of the disease can be predicted with a specificity exceeding 82%, sensitivity surpassing 86%, and an overall accuracy exceeding 84%.^94^

Regarding LC assessment, the work by Ahmad et al. (2023) analyzes the role of AI in LC management, focusing on how AI methodologies facilitate diagnostic accuracy, patient monitoring, and the understanding of LC symptoms. In this study, they state that there is an urgent need for more integrated enhanced care techniques to improve patient outcomes, through the analysis of different ML approaches, such as random forest neural and neural networks, for predicting LC prevalence based on biomarkers; they also considered information from patients’ clinical history and natural language approaches in social media. It is important to state that gathering any kind of data regarding LC is crucial for the AI-driven model’s function and training. Researchers and caregivers all over the world have been focusing on the development and application of tools that, coupled with AI methodologies, could aid in addressing this public health issue by understanding the underlying physiology, explaining heterogeneity, and identifying therapeutic targets. AI-driven chemometrics is highlighted as an increasingly evolving tool for processing complex biomedical and biochemical data that could potentially provide tools for analyzing large data sets such as electronic health records (EHRs), clinical notes, imaging, and patient demographics.^95^

**Figure 4:**
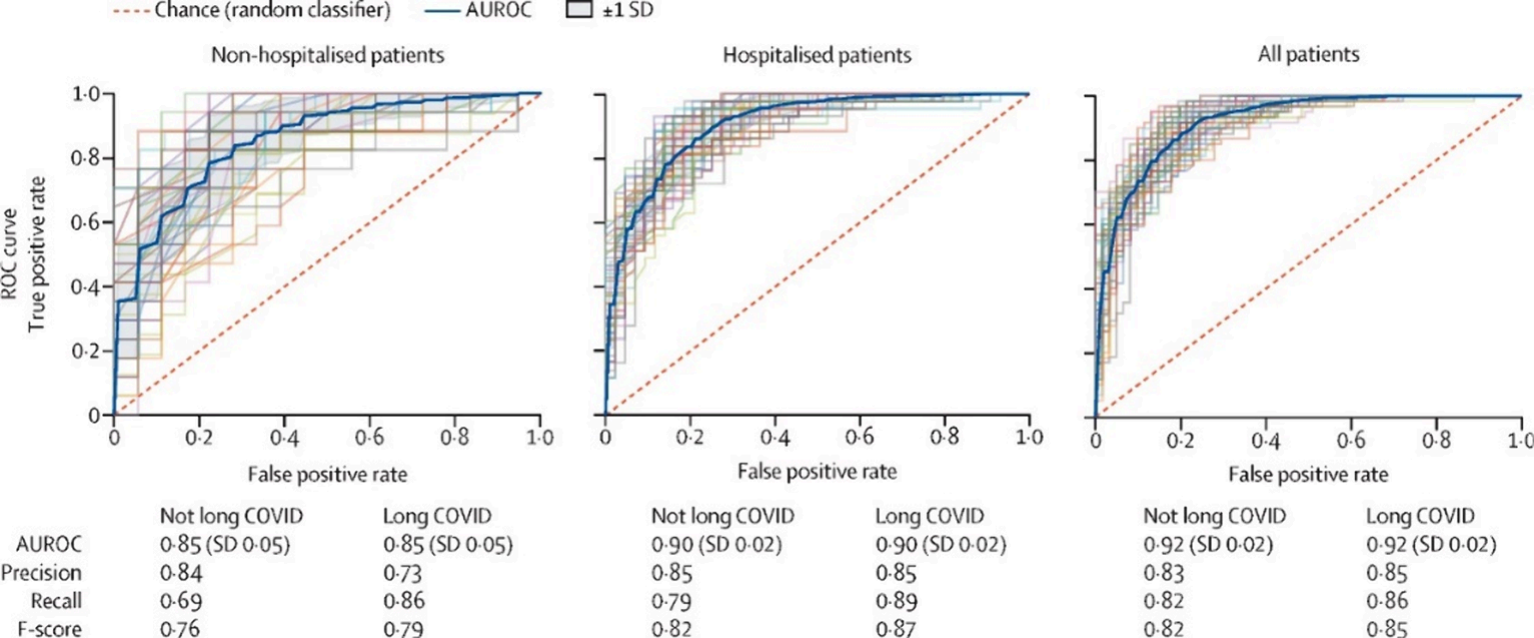
Machine learning model performance in identifying potential long COVID in patients. Reproduced from Pfaff et al., 2022, https://www.thelancet.com/journals/landig/article/PIIS2589-7500(22)00048-6/fulltext distributed with Creative Commons Attribution License (https://creativecommons.org/licenses/by/4.0/).

Pfaff et al. (2022) reported an ML algorithm based on the N3C EHR database, clinical biomarkers, patient symptoms, and demographic data for LC assessment. Researchers collected 924 features from 597 patients diagnosed with LC and trained three ML models to predict LC and discriminate LC from COVID-19 patients. Their results showed that the gradient boost (XGBoost) algorithm achieved an AUC value of 0.92 for all patients, 0.90 for hospitalized patients, and 0.85 for outpatients; the results from their model are presented in Figure 4.^96^ Also, Patel et al. (2023) studied the expression of 2925 unique blood proteins in LC outpatients compared to that in COVID-19 patients and healthy controls. ML analysis identified 119 relevant proteins for discriminating LC outpatients, with nine and five protein combinations with high sensitivity and specificity for LC (AUC = 1.00, F1 = 1.00). They concluded that the identified proteins reflected widespread organ and cell type expression. Optimal protein models and individual proteins hold the potential for accurate diagnosis of LC and targeted therapeutics for this disease.^97^

Another approach that has been published is the work by Zoodsma et al. (2022); they performed targeted proteomics to evaluate inflammatory biomarkers and cytokines in LC and COVID-19 patients and healthy controls. The results of chemometric analysis to investigate proteomic dynamics indicate that the proteome of hospitalized patients is remarkably different from that of LC or healthy individuals. In contrast, the differences between LC and healthy individuals are more subtle. They found 196 proteins, of which 30 were specific to LC, and they showed that these individual proteins are related to ongoing inflammation in this condition.^98^ A recent study by Jiang et al. (2022) focused on the association of symptoms such as oxygen levels, heartbeat, and blood pressure with LC, and through the XGBoost approach, they predicted LC outcome, and they additionally used convolutional neural networks (CNN) and long short-term memory (LSTM) to process a multidimensional time series of vital measures and validated through cross-validation.^99^ Finally, one interesting ML approach is the one reported by Zhang et al. (2023), which was made considering newly acquired medical conditions during the postacute phase of a COVID-19 infection in EHRs. Using a 137-dimensional binary encoding, patterns in LC patient data were identified, grouping recurring conditions with specific probabilities. Topic modeling was then used to represent these patterns in a lower-dimensional space, revealing four primary LC subphenotypes: (i) cardiac and renal issues (33.75% development, 25.43% validation), (ii) respiratory, sleep, and anxiety issues (32.75% and 38.48%), (iii) musculoskeletal and nervous system complications (23.37% and 23.35%), and (iv) digestive and respiratory problems (10.14% and 12.74%), each linked to distinct demographics.^100^

In the context of AI-driven chemometrics for eNose assessment of LC, the studies presented in this review above, give a context for using this technology to generate reliable chemometric algorithms to enhance their results in evaluating this condition. There is a need to further expand the use of this technology in the LC scenarios, to study the application, as well as understand the VOC patterns, and, most importantly, to tailor and validate this hybrid analytical strategy that can adapt to the unique characteristics and patient diversity in LC. It is important to mention that appropriate statistical analysis is of major importance when analyzing exhaled breath samples, especially when applying eNoses. These complex statistical approaches are often based on the following: (i) Exploratory (unsupervised) data analysis, which does not rely on prior hypotheses and commonly includes techniques like PCA or other clustering methods. Unsupervised methodologies help uncover new relationships within the data and can lead to new research questions. However, their effectiveness can be limited because dominant patterns often stem from nondisease factors such as gender, environmental influences, or comorbidities. (ii) Supervised analysis, where univariate analysis can be employed when adjusted for multiple testing. Once the data are preprocessed, multivariate techniques are applied to either the predictor matrix or a projection. Common applied supervised methods include discriminant analysis (i.e., LDA, PLS-DA, and OPLS-DA), support vector machines (SVM), neural networks (NN), decision trees, and Bayesian approaches. These techniques are used to classify and predict outcomes, making them highly effective in identifying disease-specific patterns within complex breath data. In this context, the study by Leopold et al., 2015 systematically compares various statistical approaches used for analyzing data eNoses in breath analysis. The study focuses on (i) dimension reduction, (ii) classification, and (iii) validation methods, identifying how these choices affect the diagnostic performance of eNoses, for (i) dimension reduction they reported as the most applied methodologies PCA and PLS-DA, (ii) for classification, LDA, SVM, and NN with LDA as the most used classification approach, which also provided the best results, and for (iii) validation they reported as the most used approaches, cross-validation and external validation, with performance generally decreasing when models were tested on external data sets, highlighting the importance of using external validation to avoid overfitting. This study showed that no single combination of methods consistently is efficient and external validation is crucial to avoid overoptimistic results. Each data set and study design may require different analytical techniques tailored to the characteristics of the data and patient population.^101^ In this context, applying eNoses in the LC scenario requires extensive validation and well-characterized patients to provide accurate information to healthcare providers. The diagnostic challenges associated with LC stem from its multisystemic nature, characterized by a diverse array of seemingly unrelated symptoms. Extensive research efforts have been dedicated to generating methodologies for the straightforward diagnosis of the condition. However, currently, standardized diagnostic procedures are needed. Consequently, integrating algorithms—AI-driven algorithms—, along with novel analytical techniques, emerging biomarkers, VOCs, and advanced imaging technology, becomes paramount.

This convergence aims to discern patterns of symptoms, enabling a reliable diagnosis and formulation of personalized treatments. The ultimate goal is to enhance the quality of life for individuals dealing with LC. To this extent, AI-driven chemometrics are emerging tools for physicians in decision-making, enriching daily clinical practice by providing insights into multiorgan involvement. This transformative approach could simplify diagnosis and treatment, potentially improving overall patient outcomes, giving the path of a revolutionary phase in precision medicine and enhancing healthcare practices to ground-breaking standards of efficiency and effectiveness. However, there is still great work to develop for these methods to be applied in clinical practice, such as (i) external validation on diverse and representative data sets to ensure their generalizability across different populations and settings; (ii) availability of high-quality and standardized data is crucial for training accurate AI models; (iii) interpretable models that are accessible and understandable for the healthcare personnel; (iv) integration of this methodologies into the clinical workflow; (v) clinical collaboration and user education between scientists, AI developers, and healthcare professionals that result in the effective use of these tools in the clinical practice; and (vi) and the most important challenge, the limited understanding of LC; this disease is still evolving, and ongoing research is needed to characterize the condition better and identify reliable biomarkers or diagnostic features.

In general, deploying AI-driven chemometrics in clinical practice still brings potential risks to be considered, including bias and limited generalizability if training data sets fail to represent diverse patient populations accurately. This can lead to inequitable and inaccurate predictions. Data privacy and security are also significant concerns, as clinical data are susceptible, and mishandling can breach patient trust and violate regulations. Additionally, many advanced AI models operate as “black boxes,” making it challenging for clinicians to interpret predictions, which may hinder trust and clinical decision-making. Integrating AI into healthcare systems is complex, as infrastructure and workflows differ, potentially causing disruptions in care and limiting model adoption. Regulatory challenges also pose risks, as AI models must meet compliance standards, yet regulatory frameworks for AI in healthcare are still evolving, leading to risks of premature deployment without adequate validation. Data quality is crucial, as clinical data inconsistencies can weaken AI accuracy. Over-reliance on AI outputs could lead clinicians to overlook contextual factors and clinical judgment, risking patient safety. Moreover, the rapid evolution of medical knowledge means that AI models can quickly become outdated, necessitating regular updates to maintain accuracy. Ethical and liability concerns occur when AI-driven decisions lead to adverse outcomes, creating legal and ethical challenges around accountability. Addressing these risks requires the generation of regulatory guidelines, validation processes, and continuous collaboration among AI developers, healthcare providers, and regulators to ensure safe, reliable, and ethically sound AI integration in clinical settings.

Despite the challenges, the integration of AI in clinical practice is promising, especially in addressing complex, multifaceted conditions such as LC. The potential of AI-driven technologies to provide faster, more accurate, and personalized diagnostics could revolutionize healthcare by identifying patterns and biomarkers that might be invisible to human analysis alone. The application of AI in analyzing exhaled breath VOCs offers a noninvasive manner to early detection and continuous monitoring, bringing hope to patients dealing with the enduring symptoms of LC. As we refine these models, prioritize patient privacy, and build more inclusive data sets, we move closer to realizing a healthcare system that is not only more efficient but also more equitable and patient-centered. Advances in AI are making these technologies more accessible to clinicians, promoting trust, and empowering them with guidance to enhance patient care. The integration of AI, data science, and clinical expertise could enable a future where LC and other complex diseases can be managed with precision and greater hope for improved quality of life. Through continued collaboration and innovation, AI-driven tools could transform healthcare into a more resilient and responsive system to further prevent new challenges in the emergence of new/complex diseases such as LC.

## Conclusions

Long COVID presents a complex clinical syndrome impacting a substantial global population, comprising individuals who have recovered from the acute phase of SARS-CoV-2 infection, and this condition is still a challenge for healthcare providers and authorities not only in the understanding of the disease but also with a direct impact in developing efficient therapies and enhancing the patient outcome. The persistent diverse symptoms across various organs and systems could obstruct LC diagnosis and management complexities. Despite ongoing efforts, the etiology and pathophysiology remain incompletely understood. In the field of diagnostics and patient screening, existing approaches, while valuable, face challenges in standardization and completeness; these include blood tests, imaging, pulmonary function tests, questionnaires, and telemedicine as an emerging area. To face these challenges, new technologies are being developed, among which sensor developments are emerging in clinical applications. They offer several advantages in wearable device development, enabling in-house patient care monitoring and making telemedicine a reality. One important approach is the sensor-based electronic for LC assessment, with the analysis of VOC in exhaled breath offering a non-invasive and rapid diagnostic method. Although the specific VOC associated with LC is yet to be fully characterized, insights from COVID-19 research suggest potential markers for further investigation, and some reports indicate that these biomarkers can be monitored even after the negativization and potentially proposed for LC monitoring and management along with AI-driven chemometrics. In this regard, its integration represents a revolutionary phase, with new, more sensitive, innovative, and selective sensors, DL models, and ML algorithms showing high accuracy in identifying and predicting disease outcomes. However, evidence for LC remains limited, also to encourage ongoing research and collaboration to effectively apply these tools in clinical practice, addressing challenges such as external validation, standardized data availability, interpretability, and the evolving nature of LC. Despite these challenges, the potential of these technologies to revolutionize LC evaluation, offering rapid, noninvasive, and efficient diagnostics, marks a significant step toward improved patient care and outcomes. As the world deals with the lasting impact of COVID-19, the application of evolving technologies and comprehensive diagnostic, monitoring, and therapeutic strategies is crucial for advancing LC understanding and patient care, this being crucial for the inclusion of effective strategies to be applied in clinical scenarios and to improve the quality of life of the patients suffering from this condition.

This review aims to provide recent evidence for researchers, educators, and health authorities regarding the emerging health challenge posed by LC. The goal is to contribute to informed decision-making and highlight advancements in developing and applying new sensor technologies, particularly for the study of multisystemic disease, such as LC, when addressing a wide spectrum of symptoms, which is the key to disease diagnosis and outcome.
